# Retrospective Single-Center Study on the Epidemiological Characteristics of Influenza B Infections in Korea (2007–2024): Analysis of Sex, Age, and Seasonal Patterns

**DOI:** 10.3390/microorganisms13051141

**Published:** 2025-05-16

**Authors:** Jeong Su Han, Yoo Na Chung, Jae Kyung Kim

**Affiliations:** 1 Department of Biomedical Laboratory Science, College of Health Sciences, Dankook University, Cheonan-si 31116, Republic of Korea; jshan1162@naver.com; 2 Department of Medicine, College of Medicine, Dankook University, Cheonan-si 31116, Republic of Korea; nottinghum@naver.com

**Keywords:** aged, child, hospital, human, influenza, real-time polymerase chain reaction, season, virus

## Abstract

Influenza B, a globally prevalent respiratory virus, particularly affects children, the elderly, and individuals with chronic diseases. This retrospective single-center study analyzed long-term epidemiological trends using 23,284 PCR test results from Dankook University Hospital, Cheonan-si, Republic of Korea, from 2007 to 2024. The data included inpatients and outpatients who presented with respiratory symptoms and underwent multiplex PCR testing. Unlike previous studies focusing on short-term outbreaks, this study examines extended trends and emerging seasonal patterns. Positivity rates were statistically analyzed by year, season, sex, age group, and the impact of COVID-19 (2020–2022). Significant annual differences (*p* < 0.001) occurred, with peaks in 2012 and 2018 and a sharp decline during 2020–2022. Children exhibited the highest positivity rate (2.40%), significantly higher than that of adults (2.24%) and the elderly (1.79%) (*p* < 0.05). Infections peaked in the winter (2.98%) and spring (3.95%), contrary to the belief that Influenza B peaks in winter only. Females had a higher positivity rate (2.13%) than males (1.70%) (*p* = 0.017). These findings provide novel insights into Influenza B epidemiology, emphasizing the need for prevention strategies beyond winter. The secondary spring peak suggests extending vaccination to early spring may improve influenza control, particularly among high-risk groups.

## 1. Introduction

Influenza B is an infectious respiratory disease that annually circulates worldwide, with a particularly high incidence during winter, and is transmitted between individuals via respiratory droplets [1]. Although both Influenza A and B viruses are categorized as influenza viruses, they exhibit distinct viral types, genetic compositions, and epidemic patterns. Influenza A virus is susceptible to mutations [2], typically resulting in large-scale outbreaks and pandemics, whereas Influenza B virus tends to mutate less frequently and generally causes localized epidemics [3]. However, Influenza B can lead to complications in vulnerable populations such as children, the elderly, and immunocompromised individuals [4]. Consequently, preventive measures and management strategies for these groups are crucial. Research on current epidemic trends yields essential foundational data for health policy, facilitating the formulation of precise and effective prevention and response strategies [5].

Extensive research has been conducted on the Influenza A virus, primarily owing to its frequent antigenic shifts and pandemic potential. In contrast, long-term epidemiological studies on Influenza B remain limited despite its significant impact on public health. Unlike Influenza A virus, which undergoes rapid genetic changes, Influenza B virus evolves more slowly and exhibits distinct seasonal and demographic infection patterns, necessitating a separate and detailed analysis. To address this gap, our study exclusively focuses on the Influenza B virus, providing a comprehensive long-term assessment of its epidemiological characteristics. The epidemic pattern of Influenza B varies by sex, age group, and season, necessitating a comprehensive analysis of these variables [6]. Evaluating infection rates within specific groups enables the establishment of precise vaccination and healthcare policies tailored to each population. Analyzing seasonal variations and annual trends enhances the predictability of Influenza B outbreaks and strengthens real-time response systems [7]. The real-time polymerase chain reaction (RT-PCR) technique has emerged as an essential tool for accurate diagnosis [8] and virus type and subtype identification, enabling rapid and precise detection of infections [9].

In this study, we statistically analyzed the differences in the positivity rates of Influenza B as detected by RT-PCR testing at Dankook University Hospital (Cheonan-si, Republic of Korea) from 2007 to 2024, considering variables such as sex, age, season, and year. The aim was to elucidate the epidemic patterns of Influenza B and offer critical foundational data for developing prevention and management strategies [10]. Additionally, this study contributes to optimizing vaccination policies and improving public health interventions tailored to Influenza B outbreaks. Our findings provide valuable insights into refining influenza prevention strategies, strengthening healthcare preparedness, and enhancing policy frameworks to mitigate Influenza B transmission [11].

## 2. Materials and Methods

### 2.1. Study Design and Data Collection

This study was designed as a retrospective observational investigation. A total of 23,284 individuals were included in this study, comprising 13,961 males and 9323 females. This study was conducted at Dankook University Hospital, a tertiary care teaching hospital with 921 inpatient beds located in Cheonan-si, Republic of Korea. Nasopharyngeal swab specimens were collected from inpatients and outpatients presenting with respiratory symptoms between 2007 and 2024. These samples were subjected to molecular identification tests, including multiplex real-time PCR for Influenza B. All samples were tested immediately after collection without long-term storage. Testing was performed on individuals presenting with influenza-like symptoms—such as fever, cough, sore throat, and other respiratory symptoms—or requiring differential diagnosis for respiratory infections. The test results and patient demographic information (sex, age, and test date) were retrieved from the hospital’s laboratory information system (LIS). Samples were excluded if they had missing demographic information or an inconclusive PCR result (e.g., no amplification curve or a cycle threshold [CT] value above the defined cutoff threshold).

### 2.2. Testing Procedure

Nucleic acids were extracted from respiratory specimens using the QIAamp Viral RNA Mini Kit (Qiagen, Hilden, Germany) strictly following the manufacturer’s protocol. The extracted nucleic acids were analyzed using the AdvanSure^TM^ RV-Plus Real-Time RT-PCR Kit (LG Life Sciences, Changwon-si, Republic of Korea). The kit contains specific primers and probes for each virus and was used to detect the gene of Influenza B. PCR was carried out using the SLAN real-time PCR system (LG Life Sciences, Changwon-si, Republic of Korea), with reaction conditions set according to the manufacturer’s recommendations. The interpretation of PCR results was based on the CT values provided by the manufacturer. The interpretation of positive results was performed in accordance with the manufacturer’s guidelines.

### 2.3. Data Preprocessing

Missing data, including test results, were excluded from the analysis. Age groups were categorized based on the age classification system used in Republic of Korea, following ICH (The International Council for Harmonisation of Technical Requirements for Pharmaceuticals for Human Use) guidelines: infant (0 years), child (1–19 years), adult (20–64 years), and elderly (≥65 years). Furthermore, sex and seasonal and annual variables were categorized as follows: spring (March–May), summer (June–August), fall (September–November), and winter (December–February). Subsequently, data were segregated based on the variables above.

### 2.4. Data Analysis

Chi-square (χ^2^) tests helped evaluate statistically significant differences in Influenza B positivity rates across age groups, sexes, seasons, and years. The chi-square test was chosen because the outcome variable (Influenza B positivity) and the grouping variables (age group, sex, season, and year) were all categorical. This method is commonly used to assess the independence between categorical variables and is therefore appropriate for analyzing proportional differences in positivity rates across demographic and seasonal subgroups. For each comparison, the observed frequencies were compared to the expected frequencies under the null hypothesis of no association. The expected number of positive cases in each category was calculated by multiplying the overall positivity rate by the number of individuals tested. Expected negative cases were then derived by subtracting expected positives from the total tested. These calculations were performed as part of the assumptions of the chi-square test. Statistical significance was defined as *p* < 0.05. All statistical analyses were conducted using SPSS software (version 17.0, SPSS Inc., Chicago, IL, USA).

## 3. Results

### 3.1. Annual Incidence Trends

**Figure 1:**
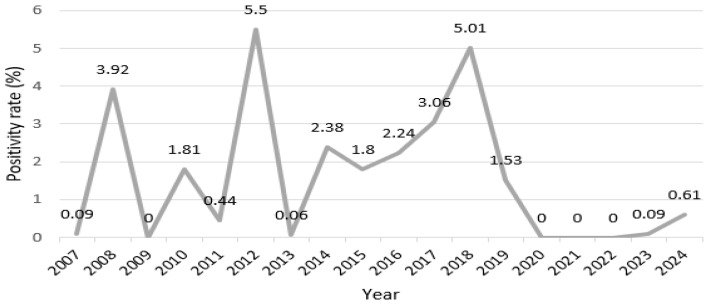
Graphical representation of annual Influenza B positivity rate fluctuations from 2007 to 2024.

In this study, we analyzed the annual positivity rates of Influenza B from 2007 to 2024 (Figure 1, Table S1) to evaluate the presence of statistically significant differences in positivity rates over time. As shown in Table S1, the number of tested individuals, positive cases, and calculated positivity rates varied considerably year by year. The analysis revealed statistically significant differences in the annual positivity rates for Influenza B (*p* < 0.001). Specifically, 2012 and 2018 recorded remarkably higher positivity rates than other years. However, from 2020 to 2022, the positivity rate was 0%, depicting notably low values.

### 3.2. Seasonal Patterns

To analyze significant differences in the positivity rates of Influenza B by season, we examined the positivity and negativity data for each season from the 23,284 samples included in the study. Seasonal data (Table 1) revealed that, during spring (March–May), summer (June–August), fall (September–November), and winter (December–February), 243 out of 6391, 5 out of 4810, 1 out of 5607, and 188 out of 6476 individuals had positive results, respectively.

The analysis revealed a statistically significant difference in the positivity rates of Influenza B between seasons (*p* < 0.001). Seasonal patterns revealed that the positivity rates for Influenza B were relatively high during spring and winter. Specifically, during spring, 243 out of 6391 individuals tested positive, resulting in a positivity rate of approximately 3.95%, whereas during winter, 188 out of 6476 individuals were positive, with a positivity rate of approximately 2.98%. Contrastingly, summer (positivity rate: 0.10%) and fall (positivity rate: 0.01%) exhibited negligible positivity rates.

Based on the monthly distribution of Influenza B-positive cases, the seasonal pattern varied considerably across the study period. A spring peak was observed in 2010, 2012, 2015, 2016, and 2019, whereas a clear winter peak occurred in 2017 and 2018. In 2011 and 2024, a slight increase in positive cases was noted during winter, suggesting weak seasonal activity rather than pronounced peaks. Dual seasonal peaks, one in spring and another in winter, were identified in 2008 and 2014. These findings indicate that Influenza B does not follow a fixed seasonal pattern but exhibits year-to-year circulation variability.

### 3.3. Sex Analysis

This study analyzed the differences in positivity rates for Influenza B based on sex using test data from 2007 to 2024. Among the 23,284 individuals tested, 13,961 were male and 9323 were female. Of these, 238 males and 199 females tested positive for Influenza B (Table 2).

The expected number of positive and negative cases was calculated for males and females to compare the differences in positivity rates between sexes. The expected number of positive and negative male cases was approximately 262.02 and 13,698.97, respectively. For females, the expected number of positive and negative cases was approximately 174.97 and 9148.02, respectively. The chi-square test indicated a significant difference in positivity rates between males and females (χ^2^ = 5.70, *p* = 0.017), with females showing a higher rate.

The results indicate a statistically significant difference in Influenza B positivity rates between sexes (*p* = 0.017). The chi-square value also exceeded the critical value, further confirming significance. These results suggest that sex may be crucial in susceptibility to Influenza B, with females being more vulnerable than males. To determine whether age influenced the observed sex-based differences in positivity rates, we analyzed the age distribution among male and female positive cases (Table 3). The distribution was generally comparable: in both groups, over 60% of positive cases occurred in the 1–19 age group. This suggests that the sex difference in positivity rates was not substantially confounded by age. This highlights the need for sex-specific approaches in future infection prevention and management strategies.

### 3.4. Age Group Differences

The positivity rates for the Influenza B virus in Republic of Korea from 2007 to 2024 were analyzed by age group, classified as follows: infant (0 years), child (1–19 years), adult (20–64 years), and elderly (65 years and older). The total number of individuals tested in each age group and the number of those confirmed positive for the Influenza B virus are presented in Table 4.

The analysis of Influenza B positivity rates by age group revealed a statistically significant difference (*p* < 0.001), as the observed chi-square value (χ^2^ = 63.10) exceeded the critical value (χ^2^ = 7.81, df = 3), indicating meaningful variation in positivity rates across age groups.

Infants showed relatively low positivity rates, whereas children exhibited high positivity rates. Adults and the elderly had similar positivity rates, although the elderly group demonstrated a relatively lower positivity rate than other adult groups. Future preventive measures for Influenza B should involve age-specific strategies tailored to the needs of each group. From 2007 to 2017, most Influenza B-positive cases occurred in the 1–19 age group, accounting for over 60% of all positive cases and indicating a consistent pediatric predominance. However, in 2018, a notable shift was observed, with more than 50% of cases reported in individuals aged 65 and older (Table S2). Although lineage-level data were unavailable, this age-related shift may reflect underlying changes in circulating virus strains or other external factors.

## 4. Discussion

This study provides a comprehensive analysis of the long-term epidemiological patterns of Influenza B in Korea over 18 years, focusing on temporal, seasonal, sex-based, and age-specific trends. Unlike previous studies that were limited to short-term surveillance, our findings reveal distinct patterns in transmission that offer valuable implications for public health preparedness. Influenza B positivity varied significantly across the years, with some periods showing elevated detection and others marked by a near-absence of cases. Various factors likely influenced these interannual differences, including herd immunity levels, viral activity, and social-environmental conditions [12]. For instance, the drastic reduction in Influenza B detection during the COVID-19 pandemic aligns with implementing public health interventions such as mask-wearing, social distancing, and school closures, effectively reducing transmission [13,14,15]. These observations highlight the impact of behavioral and policy-level changes on respiratory virus dynamics [16]. Due to varying test volumes by year, caution should be exercised when interpreting positivity rates, particularly in years with low sample counts. Seasonal analysis revealed that Influenza B activity was predominantly concentrated in colder months, particularly winter and spring. This pattern is consistent with the known biology of the virus, where low temperatures and reduced humidity enhance viral stability and transmissibility, while increased indoor activity fosters closer contact [17,18]. Spring peaks may also be associated with heightened social interactions and school reopening periods [19]. Conversely, higher temperatures and humidity in summer and fall likely suppress viral transmission [20,21], while increased sun exposure during summer could elevate vitamin D levels and enhance immune defense [22]. The seasonal distribution of Influenza B showed substantial variation across the years. Spring-dominant peaks were observed in 2010, 2012, 2015, 2016, and 2019, whereas winter-dominant peaks were more prominent in 2017 and 2018. In 2011 and 2024, a slight increase in positive cases was noted during winter, suggesting weak seasonal activity rather than pronounced peaks. In this study, a ‘dominant peak’ refers to the season during which the highest number of Influenza B-positive cases occurred in a given year. In 2008 and 2014, dual seasonal peaks occurred in both spring and winter. In this study, the observed year-to-year variability in peak timing and intensity emphasizes the importance of implementing a flexible surveillance system. This system should have the capability to dynamically adjust testing frequency and targeted populations (e.g., children or older adults) and allocate resources accordingly based on emerging patterns. For example, earlier testing efforts may be required in years when transmission begins in the late fall or unexpectedly shifts from winter to spring. This approach enhances early detection, enables proactive intervention, and prevents under- or over-utilization of resources. These findings highlight the importance of implementing flexible surveillance strategies that can accommodate year-to-year fluctuations in seasonal trends. Notably, seasonal peaks varied between years: some exhibiting spring-dominant activity, others winter-dominant, and a few displaying dual seasonal peaks. This year-to-year variability highlights the importance of flexible surveillance strategies that can adapt to evolving seasonal trends. Regarding sex-based differences, this study observed a statistically significant disparity in positivity rates between males and females. Although a slightly higher positivity rate was observed in females, the biological or behavioral basis for this sex-based disparity remains unclear. As few previous studies have directly examined sex-based differences in Influenza B positivity rates, our interpretation is limited to the observed statistical findings. Further research is needed to investigate biological, behavioral, or environmental factors contributing to this disparity. In 2018, the number of Influenza B-positive cases among individuals aged ≥65 was the same for both sexes (24 men and 24 women), indicating that the observed increase in older female cases was not caused by any disparity in sample size. However, in 2018, there was a noticeable winter peak, and this seasonal factor may have contributed to the increased positivity in the elderly [23]. These findings suggest that considering sex-specific factors may be beneficial in future infection prevention and surveillance strategies. Previous literature has rarely addressed Influenza B positivity from a sex-specific perspective, highlighting the importance of our findings as a potential direction for future research. It remains essential to consider potential physiological, hormonal, and social behavioral factors contributing to sex-based differences in infection dynamics. Regarding age distribution, the highest positivity rates were consistently observed among children and adolescents, reaffirming their role as a key reservoir and driver of community transmission. This aligns with immunological studies suggesting that younger individuals, particularly those with immature or developing immune systems, are more susceptible to respiratory infections [24,25]. In contrast, adults and the elderly generally showed lower positivity rates. However, a notable age shift occurred in a specific year when elderly individuals represented a disproportionate share of cases. This raises the possibility of virus characteristics or host susceptibility changes, which could be linked to shifts in circulating lineages [26,27,28]. Although most cases consistently occurred in the pediatric population from 2007 to 2017, a notable increase in elderly cases was observed in 2018. This may indicate age-related shifts in susceptibility, potentially influenced by changes in circulating viral lineages. Future studies incorporating genetic or lineage-level data are warranted to investigate this trend further. Despite its strengths, it is important to acknowledge the various limitations of this study. First, the analysis was confined to a single tertiary hospital in Korea, which may limit the generalizability of the findings to other regions. Second, important external factors such as vaccination coverage, climate variability, and changes in virus genotype were not fully accounted for, despite their potential to greatly influence Influenza B transmission dynamics. Third, the absence of genomic data prevented lineage-specific analysis, which could have provided insight into some of the observed trends. Future research should incorporate multivariate models, genomic sequencing, and longitudinal cohort designs to comprehensively investigate the factors affecting Influenza B transmission. These insights will be valuable for informing public health decision-making, including vaccination scheduling, school health policies, and elderly care strategies. Overall, this study provides valuable long-term epidemiological insights into Influenza B infections and emphasizes the need for seasonally adaptive age- and sex-specific public health interventions.

## 5. Conclusions

This study provides long-term epidemiological insights into Influenza B infections in Korea from 2007 to 2024, highlighting significant variations in positivity rates by year, season, sex, and age group. Unlike previous studies focusing on short-term outbreaks, this study reveals persistent seasonal and demographic patterns, offering valuable insights for developing influenza prevention strategies. Our findings suggest that Influenza B infections were most prevalent in children and peaked during winter and spring, whereas positivity rates were significantly low in summer and fall. The sharp decline in positivity rates during the COVID-19 pandemic (2020–2022) suggests that public health interventions, such as social distancing and mask mandates, played a crucial role in reducing transmission. Additionally, females exhibited a higher positivity rate than males, likely owing to immunological and hormonal differences. The significantly higher positivity rate in children (2.40%) than in adults and the elderly highlights the necessity of targeted vaccination programs for pediatric populations. Considering the decline in immune function with age, continued vaccination efforts for older adults remain essential in mitigating Influenza B-related morbidity and mortality.

To effectively mitigate Influenza B transmission, the following public health measures should be considered: (1) Expansion of vaccination programs: Given the high positivity rates in winter and spring, influenza vaccination should focus on pre-winter immunization, as well as consider booster doses in early spring to mitigate secondary outbreaks. (2) Targeted infection control measures: Schools, childcare centers, and elderly care facilities should reinforce prevention strategies such as seasonal mask-wearing recommendations, hand hygiene campaigns, and improved ventilation, particularly from late fall to early spring. (3) Enhanced surveillance and early warning systems: Expanding influenza surveillance programs to include real-time monitoring of seasonal trends and genomic analysis of circulating strains could improve outbreak predictions and facilitate timely public health responses. (4) Targeted public health campaigns: Increasing awareness about timely vaccination and personal preventive measures, particularly among high-risk groups such as children, the elderly, and individuals with chronic illnesses, can further enhance influenza control efforts. By implementing these targeted strategies, public health agencies can significantly reduce Influenza B transmission and improve population health outcomes. Future studies should incorporate genomic surveillance data to track viral mutations and assess their impact on vaccine effectiveness across different demographic groups, ensuring effective prevention and management strategies for seasonal Influenza B outbreaks. By utilizing the findings of this study, health authorities can more effectively allocate resources, adjust vaccination schedules, and prepare targeted interventions for future seasonal Influenza B outbreaks.

## Figures and Tables

**Table 1:** Influenza B seasonal positivity rates from 2007 to 2024.

Season	Positive	Negative	Positivity Rate (%)
Spring	243	6148	3.95
Summer	5	4805	0.10
Autumn	1	5606	0.01
Winter	188	6288	2.98

**Table 2:** Comparison of Influenza B positivity rates by sex from 2007 to 2024.

Sex	Positive	Negative	Positivity Rate (%)
Male	238	13,723	1.70
Female	199	9124	2.13

**Table 3:** Age distribution of Influenza B-positive cases by sex.

Age Group	Male (*n* = 238)	Female (*n* = 199)
Infants (0 years)	13 (5.5%)	10 (5.0%)
Children (1–19 years)	144 (60.5%)	121 (60.8%)
Adults (20–64 years)	39 (16.3%)	26 (13.1%)
Older adults (65 years and above)	42 (17.6%)	42 (21.1%)

**Table 4:** Influenza B positivity rates by age group from 2007 to 2024.

Age Group	Total Individuals	Positive	Negative	Positivity Rate (%)
Infant (0 years)	4556	23	4533	0.50
Child (1–19 years)	11,137	265	10,872	2.40
Adult (20–64 years)	2899	65	2834	2.24
Elderly (65 years and above)	4692	84	4608	1.79

## Data Availability

The data supporting the findings of this study are not publicly available due to institutional policy but may be provided upon reasonable request and with the approval of the corresponding author.

## Supplementary Materials

The following supporting information can be downloaded at: https://www.mdpi.com/article/10.3390/microorganisms13051141/s1, Table S1: Annual Influenza B testing summary: Number of tested individuals, positive cases, and positivity rates from 2007 to 2024. Table S2: Annual Influenza B positive cases and age-specific proportions in Republic of Korea (2007–2024).

## Abbreviations

The following abbreviations are used in this manuscript:

RT-PCR	Real-time polymerase chain reaction
CT	Cycle threshold
