# Supplementary material for: Retrospective Single-Center Study on the Epidemiological Characteristics of Influenza B Infections in Korea (2007–2024): Analysis of Sex, Age, and Seasonal Patterns
Source: Microorganisms. 2025 May 16;13(5):1141. doi: 10.3390/microorganisms13051141 (PMC12113934; doi:10.3390/microorganisms13051141)
Supplement: Supplementary file 1 [file microorganisms-13-01141-s001.zip › microorganisms-3541946-supplementary.pdf]

Table S1. Annual Influenza B testing summary: Number of tested individuals, positive cases, and positivity rates from 2007 to 2024.

Year	Total individuals	Positive	Positivity rate (%)
2007	1,057	1	0.09
2008	1,504	59	3.92
2009	1,265	0	0
2010	1,657	30	1.81
2011	1,568	7	0.44
2012	1,345	74	5.5
2013	1,545	1	0.06
2014	1,674	40	2.38
2015	1,388	25	1.8
2016	1,645	37	2.24
2017	1,436	44	3.06
2018	1,834	92	5.01
2019	1,432	22	1.53
2020	792	0	0
2021	613	0	0
2022	860	0	0
2023	1,016	1	0.09
2024	653	4	0.61

Table S2. Annual Influenza B positive cases and age-specific proportions in Republic of Korea (2007–2024)

Year	Age(0)	Age(1-19)	Age(20-64)	Age(65+)	Total	%Age(0)	%(1-19)	%(20-64)	%(65+)
2007	0	1	0	0	1	0.0	100.0	0.0	0.0
2008	3	42	7	7	59	5.1	71.2	11.9	11.9
2009	0	0	0	0	0	0.0	0.0	0.0	0.0
2010	1	28	0	1	30	3.3	93.3	0.0	3.3
2011	0	4	2	1	7	0.0	57.1	28.6	14.3
2012	4	52	8	10	74	5.4	70.3	10.8	13.5
2013	0	1	0	0	1	0.0	100.0	0.0	0.0
2014	4	19	8	9	40	10.0	47.5	20.0	22.5
2015	1	20	3	1	25	4.0	80.0	12.0	4.0
2016	4	28	5	0	37	10.8	75.7	13.5	0.0
2017	5	23	8	8	44	11.4	52.3	18.2	18.2
2018	1	25	20	46	92	1.1	27.2	21.7	50.0
2019	0	21	1	0	22	0.0	95.5	4.5	0.0
2020	0	0	0	0	0	0.0	0.0	0.0	0.0
2021	0	0	0	0	0	0.0	0.0	0.0	0.0
2022	0	0	0	0	0	0.0	0.0	0.0	0.0
2023	0	1	0	0	1	0.0	100.0	0.0	0.0
2024	0	0	3	1	4	0.0	0.0	75.0	25.0
